# Early detection of microstructural white matter changes associated with arterial pulsatility

**DOI:** 10.3389/fnhum.2013.00782

**Published:** 2013-11-18

**Authors:** Todd A. D. Jolly, Grant A. Bateman, Christopher R. Levi, Mark W. Parsons, Patricia T. Michie, Frini Karayanidis

**Affiliations:** ^1^Functional Neuroimaging Laboratory, Faculty of Science and IT, School of Psychology, University of NewcastleNewcastle, NSW, Australia; ^2^Centre for Translational Neuroscience and Mental Health Research, University of NewcastleNewcastle, NSW, Australia; ^3^Hunter Medical Research InstituteNewcastle, NSW, Australia; ^4^Faculty of Health, School of Medicine and Public Health, University of NewcastleNewcastle, NSW, Australia; ^5^Department of Medical Imaging, Newcastle Region Mail Centre, John Hunter HospitalNewcastle, NSW, Australia; ^6^Hunter Stroke Service, Hunter New England HealthNewcastle, NSW, Australia

**Keywords:** arterial pulsatility, pulse wave encephalopathy, leukoaraiosis, fractional anisotropy, diffusion tensor imaging

## Abstract

Increased cerebral blood flow pulsatility is common in vascular dementia and is associated with macrostructural damage to cerebral white matter or leukoaraiosis (LA). In this study, we examine whether cerebral blood flow pulsatility is associated with macrostructural and microstructural changes in cerebral white matter in older adults with no or mild LA and no evidence of dementia. Diffusion Tensor Imaging was used to measure fractional anisotropy (FA), an index of the microstructural integrity of white matter, and radial diffusivity (RaD), a measure sensitive to the integrity of myelin. When controlling for age, increased arterial pulsation was associated with deterioration in both measures of white matter microstructure but not LA severity. A stepwise multiple linear regression model revealed that arterial pulsatility index was the strongest predictor of FA (*R* = 0.483, adjusted *R*^2^ = 0.220), followed by LA severity, but not age. These findings suggest that arterial pulsatility may provide insight into age-related reduction in white matter FA. Specifically, increased arterial pulsatility may increase perivascular shear stress and lead to accumulation of damage to perivascular oligodendrocytes, resulting in microstructural changes in white matter and contributing to proliferation of LA over time. Changes in cerebral blood flow pulsatility may therefore provide a sensitive index of white matter health that could facilitate the early detection of risk for perivascular white matter damage and the assessment of the effectiveness of preventative treatment targeted at reducing pulsatility.

## Introduction

Cerebral white matter changes are prevalent in healthy ageing and are typically detected as leukoaraiosis (LA), a common radiological finding that presents as increased signal intensity on T2-weighted MR images. These macrostructural changes in cerebral white matter are generally thought to result from inadequate arterial flow and resultant ischaemia. However, cerebral blood flow pulsatility may also contribute or indeed be the primary contributor to these macrostructural white matter changes (Bateman et al., 2006; Mitchell et al., 2011). In order to establish whether cerebral blood flow pulsatility may be a useful indicator of emerging white matter pathology in otherwise asymptomatic individuals, we examined the association between blood flow pulsatility and microstructural properties of white matter measured using Diffusion Tensor Imaging (DTI). This could, ultimately, have implications for early detection of emerging white matter damage and guide interventions to stem the development of LA.

Arterial flow is pulsatile and causes a periodic increase in blood volume entering the intracranial cavity. According to the Monroe-Kellie doctrine, since the intracranial cavity has a fixed volume that is confined by a rigid skull, volume expansion due to arterial systolic flow must be accompanied by an equal volume reduction via the ejection of cerebrospinal fluid (CSF) and/or venous flow out of the cranial cavity. Given that physiological capillary flow is non-pulsatile, blood flow into the cranial cavity of a greater pulsatility will contain more energy that needs to be dissipated prior to reaching the capillary bed. Under normal physiological conditions, this pulsation energy is dampened via CSF and venous flow out of the cranial cavity, a phenomenon known as the windkessel effect. Normal ageing and dementia have been linked to a breakdown in the windkessel effect, resulting from an increase in the magnitude of arterial pulsations and/or a reduction in compliance of outflow pathways. The tissue damage that may result from such abnormal arterial/venous pulsatility has been termed pulse wave encephalopathy (Bateman, 2002).

Presence of LA has been associated with cognitive and motor decline in both healthy ageing and vascular dementia (Schmidt et al., 1999; Tullberg et al., 2004; Paul et al., 2005; Sachdev et al., 2005; van der Flier et al., 2005; Onen et al., 2008). While LA onset has traditionally been thought to result directly from brain ischaemia, many studies show no significant relationship between LA and rate of cerebral arterial inflow (Bateman, 2002; Henry-Feugeas et al., 2009) or carotid flow (Patankar et al., 2006). Instead, LA has been quite strongly associated with abnormal cerebral blood flow pulsations (Bateman, 2002; Doepp et al., 2006; Bateman et al., 2008; Henry-Feugeas et al., 2009; Mitchell et al., 2011; Webb et al., 2012). Several studies have shown that cerebral pulsations are positively associated with severity of LA (de Leeuw et al., 2000; Webb et al., 2012). For example, Bateman (2002) found that arterial pulsatility increased progressively with increasing LA in patients with idiopathic dementia. Webb et al. (2012) identified a similar relationship between arterial pulsatility and LA in patients who had recently suffered a transient ischaemic attack or mild stroke, with increased middle cerebral artery pulsatility corresponding with an increase in LA severity. Patients with vascular dementia also show much higher middle cerebral artery and internal carotid pulsatility when compared to normal age-matched controls (Biedert et al., 1993; Doepp et al., 2006; Vicenzini et al., 2007). Moreover, Bateman et al. (2008) showed that both arterial and venous pulsatility are elevated in patients with LA and probable early vascular dementia.

These increases in arterial and venous pulsations were interpreted as markers for arteriosclerosis and venous collagenosis, respectively. Henry-Feugeas and Koskas (2012) outline a probable mechanism for arterial and venous remodeling and how this leads to increased pulsations and associated LA. Existing studies have examined the relationship between vascular pulsatility and LA in patients with moderate to marked LA severity (Bateman, 2002) and in patients with early signs of dementia (Bateman et al., 2008). However, it has not been investigated whether measures of arterial or venous pulsatility are useful indicators of emerging damage to white matter structure.

In the present study, we examine whether subtle damage to the white matter from pulse wave injury is detectable in older adults with mild or no LA and no evidence of dementia. In addition to measuring LA severity, we use DTI, a measure sensitive to the microstructural organization of cerebral white matter. DTI measures the degree of directional dependence of microscopic diffusion of water molecules. Since the white matter is highly organized into tracts, diffusion across these tracts is obstructed resulting in anisotropic diffusion predominantly along the orientation of fiber tracts. Fractional anisotropy (FA) is a measure of the degree to which diffusion is anisotropic and is interpreted as an index of microstructural integrity of white matter (Basser and Jones, 2002). Even microstructural changes to the white matter that affect the level of myelination and the composition of white matter bundles can be detected as a reduction in FA. Animal studies have shown that changes in directional diffusivities provide information regarding underlying changes to the white matter microstructure. Specifically, radial diffusivity (RaD) increases as a function of myelin breakdown whereas axial diffusivity is sensitive to axonal damage (Song et al., 2003, 2005; Sun et al., 2006).

To determine whether increased pulsatility is related to microscopic white matter changes indicative of emerging tissue injury, we investigate the relationship between pulsation measures from CSF, arterial and venous systems and measures of both macroscopic (LA severity) and microscopic (FA and RaD) properties of the white matter in older participants with mild or no LA and no evidence of dementia. We examine whether changes in vascular pulsatility provide a sensitive measure of microscopic changes in the white matter that emerge well before evidence of extensive LA or associated vascular dementia. If pulse wave injury results from increased perivascular shear stress and damage to oligodendrocytes, we expect that microstructural properties of white matter will be more sensitive to this injury than gross macrostructural changes. Therefore, we predict that greater arterial and venous pulsatility will be strongly associated with lower FA and higher RaD, even in the absence of substantial LA.

## Materials and methods

### Participants

Seventy (35 males) individuals aged 43–82 years were recruited between January 2011 and August 2012 with equal numbers from the Hunter Medical Research Institute (HMRI) “healthy” Volunteer Register and from a general neurology clinic. Participants recruited from the HMRI volunteer register had no history of any neurological disorder while participants who were referred by two neurologists (CL, MP) have been investigated for mild stroke or a transient ischaemic attack and had a previous routine MRI scan as part of their clinical work-up.

Five participants were excluded from analysis because they scored less than 22 (out of 30) on the Montreal Cognitive Assessment (MoCA) as this is indicative of probable dementia (Nasreddine et al., 2005). Another four participants were excluded because their MRI showed evidence of moderate to severe LA, i.e., LA volume that exceeded 1.5% of intracranial volume. An additional participant was excluded because of marked bradycardia (37 bpm) which yielded a very large pulsation magnitude.

The final group of 60 participants exhibited nil to mild LA (0.306 ± 0.338 % of intracranial volume; see Table 1) and no other neurological diagnosis. Over half (56%) presented one or more vascular risk factors for which they received appropriate medication. Our sample showed moderately high scores on the MoCA (26.74 ± 2.42). All participants also completed a comprehensive neuropsychological assessment focussing on memory and executive function, as well as a testing session using complex experimental paradigms that assess cognitive control. These data are not included in this present paper.

**Table 1:** **Mean (standard deviation) for age, Montreal Cognitive Assessment (MoCA), leukoaraiosis severity (LA), mean fractional anisotropy (FA), and radial diffusivity (RaD) as well as clinical profile of participants**.

**Measure**	**Mean (*SD*)**	**Range**
Age	65.67 years (9.31)	43–82 years
MoCA	26.74 (2.42)	22–30
LA (% Intracranial volume)	0.306 (0.338)	0.017–1.297
FA	0.398 (0.017)	0.356–0.437
RaD	0.451 (0.026)	0.406–0.515
**Clinical profile**	**Yes**	**No**
Vascular risk factors present	32 (53%)	28 (47%)
Hypertension	23 (38%)	
Hypercholesterolemia	18 (30%)	
Atrial fibrillation	7 (12%)	
Multiple vascular risk factors	19 (32%)	

### Imaging protocols

All participants were imaged on a 3T Siemens Verio scanner using a 32 channel head coil. T1-weighted images were acquired in the sagittal plan using ultrafast gradient echo 3D sequence (MPRAGE) with 1 mm isotropic voxel resolution (repetition time = 1500 ms; echo time = 2.57 ms; inversion time = 900 ms; flip angle = 9°; slice thickness = 1 mm with no gap; *TA* = 3 min 29 s). A 3D Fluid Attenuated Inversion Recovery (FLAIR) sequence with 1 mm isotropic voxels was acquired in the sagittal plane using a phase encoding acceleration factor of 2 (*TR* = 5000 ms; *TE* = 395 ms; *TI* = 1800 ms; 160 slices with slice thickness = 1 mm, *TA* = 5 min 52 s). Diffusion weighted images (DWI) were acquired in axial plane using a twice refocussed spin echo sequence with a phase encoding acceleration factor of 3 (128 × 128 matrix; *TR* = 11200 ms; *TE* = 111 ms; 55 slices with a slice thickness = 2.2 mm; *TA* = 13 min 6 s). Diffusion was measured in 64 non collinear directions with a *b*-value of 3000 mm^2^/s along with one non diffusion weighted (*b* = 0) image. This high *b*-value provides enhanced contrast to noise and decreased *T*2 shine through effect at a relative cost on signal to noise ratio (SNR) (DeLano et al., 2000). However, higher SNR's achieved at acquiring image at 3T compensates for the inferior SNR at high *b*-value DWI.

All measurements of blood flow were quantified using a retrospectively cardiac-gated phase contrast flow quantification sequence (*TR* = 26.5 ms; *TE* = 6.9 ms; slice thickness = 5 mm; matrix 256 × 256). For the quantification of arterial flow, a single excitation with a velocity encoding (venc) value of 75 cm/s was used and a section plane was implemented to intersect both the basilar artery as well as the cavernous portion of the internal carotid arteries at the level of the skull base (see Bateman, 2002). Two excitations with a venc value of 40 cm/s were used to measure the superior sagittal sinus (SSS) and straight sinus (ST) flow with the slice positioned at an angle that intersected the SSS ~2 cm above the torcular and directly through the midpoint of the ST (Bateman, 2002). To measure the flow of CSF through the cerebral aqueduct, a venc value of 22 cm/s was used with a slice placed through the lower portion of the midbrain. Any aliasing that was present was excluded by retrospectively manipulating the base lines of each resultant graph, which gave an effective upper limit of 80 cm/s for venous and 150 cm/s for arterial flows. The MR flow quantification sequence utilized by this study is available on the majority of commercial scanners.

### Quantifying measures of leukoaraiosis severity, radial diffusivity and fractional anisotropy

A FLAIR was used for the calculation of LA severity. *LA severity* was determined for each participant by manually segmenting areas of hyperintense white matter present in the FLAIR images and saving these as regions of Interest (ROI). The total volume of LA was expressed as a percentage of intracranial volume. White matter FA and RaD measures were calculated from diffusion weighted images using the software package FSL. To isolate white matter from the rest of the brain tissue, we utilized a white matter mask based on the T1 structural image in conjunction with a second mask that was derived from performing probabilistic whole brain tractography using the software package MRtrix (Tournier et al., 2012). To ensure that the measures of FA and RaD were not affected by gray matter partial volume effects, we used a threshold of 0.02% on the tractography-based mask as this threshold excluded the white/gray matter boundary regions.

### Calculating measures of cerebral blood flow

A region of interest was placed around the basilar and carotid arteries in each participant; the sum of the flow of this region gave the total *arterial inflow*. The *arterial pulsatility index* was derived by dividing the difference between maximum and minimum flow by the mean flow across this region. *Arterial pulse volume* represents the degree to which the arterial tree expands in systole and was calculated by multiplying the mean increase of flow during systole by the time over which systole occurred (see Bateman, 2002). *Arterial systole duration* was defined as the period from where the flow rate was greater than the overall mean flow rate. Since the arterial pulse volume is calculated by taking the product of the increased flow and duration of systole, it is possible for pulse volume to be within normal limits even in the presence of an abnormal increase in flow pulsatility, given that the pulse duration is shortened to an extent that will counterbalance the abnormal increase in systolic flow. Pulse volume is also affected by the length of the cardiac cycle, with longer cycles having longer systole duration, resulting in larger pulse volumes. So instead using arterial flow and arterial systole duration to calculate absolute pulse volume, we will use these to extract information regarding arterial pulse wave amplitude and pulse width. The arterial pulse amplitude and pulse width informs us about the dynamic pulse pressure changes that occur across the heartbeat and the amount of energy present in the arterial pulsation. The *arterial pulse wave amplitude* was calculated by expressing the difference between the maximal arterial and mean arterial flow as a proportion of the mean arterial flow. *Arterial pulse width* was derived by dividing arterial systole duration by the duration of the overall cardiac cycle. Measures of mean outflow, venous pulsatility as well as pulse volume, amplitude and width were calculated for the SSS and ST using the same principles as those used for the arterial measures. The *aqueduct pulse* represents the volume of CSF vented through the mesencephalic duct and was obtained for each participant by measuring the mean flow directed inferiorly during systole and multiplying it by the time taken for systole. We also measured a*queduct pulse volume* as a percentage of arterial pulse volume to derive an index of *intracranial compliance*, with an increase in percentage indicating reduction in compliance.

All flow measures were baseline corrected through the use of background subtraction. This reduces the phase offset error that occurs as a result of local magnetic field inhomogeneities. Care was taken to ensure that the baseline reference region of interest was placed in close vicinity to the vessel of interest and that it did not include any vessels or air. For CSF flow, we used a reference region of interest of 1 cm^2^ on the brain stem. For arterial and venous flow, we used a 1.5 cm^2^ reference region of interest on the brain stem and on deep white matter, respectively.

The relationship between non-pulsatile and pulsatile haemodynamic measures and measures of white matter disruption were analyzed using Pearson product-moment correlations with one-tailed α = 0.05, as hypotheses about the relationship between haemodynamic measures and white matter measures are directional. Since multiple correlations were performed, family-wise error rate was controlled using a Bonferroni correction of α/9, yielding a significance level of α = 0.005. Correlations that remained significant after Bonferroni correction were corrected for age using partial correlations, as age is also associated with changes in white matter structure. As a rule, including age as a partial factor reduced the strength of significant correlations but did not markedly change the pattern of results. Stepwise multiple linear regression models (*p* < 0.05 for entry and *p* > 0.10 for removal) were used to select whether arterial pulsatility, LA severity and age predicted mean FA.

## Results

Descriptive statistics for all pulsatile and non-pulsatile flow data are presented in Table 2. There were no significant correlations between measures of non-pulsatile blood flow (both arterial and venous) and measures of white matter disruption after correcting for age (all *p* > 0.05). Aqueduct pulse volume was not related to LA severity (*p* > 0.10) but was significantly correlated with both FA (*r* = −0.404, *p* = 0.012 after Bonferroni correction) and RaD (*r* = 0.470, *p* = 0.001 after Bonferroni correction), and these correlations remained significant after correcting for age.

**Table 2:** **Means and standard deviations for both pulsatile and non-pulsatile flow measures. SSS, superior sagittal sinus; ST, straight sinus**.

**Measure**	**Mean (*SD*)**	**Range**
**NON-PULSATILE FLOW**		
Arterial inflow (mL)	709.24 (135.20)	475.56–1158.00
SSS outflow (mL)	319.78 (68.76)	196.42–560.90
ST outflow (mL)	114.73 (25.96)	(53.82–169.46)
SSS outflow (%inflow)	45.39 (6.84)	(33.26–64.34)
ST outflow (%inflow)	16.37 (3.42)	(8.55–25.08)
**PULSE VOLUME (μL)**		
Aqueduct	55.01 (31.94)	(8.67–215.97)
Arterial	1439.77 (469.69)	(543.87–2494.73)
SSS	315.67 (165.98)	(69.84–802.98)
ST	86.57 (47.21)	(18.32–235.20)
Intracranial compliance	3.82 (1.68)	(1.10–10.81)
**PULSATILITY INDEX**		
Arterial	1.00 (0.23)	(0.61–1.56)
SSS	0.55 (0.19)	(0.22–1.18)
ST	0.46 (0.18)	(0.16–1.12)
**PULSE WAVE AMPLITUDE**		
Arterial	0.59 (0.15)	(0.32–1.05)
SSS	0.29 (0.12)	(0.11–0.69)
ST	0.24 (0.12)	(0.08–0.65)
**PULSE WIDTH**		
Arterial	0.41 (0.04)	(0.31–0.49)
SSS	0.48 (0.06)	(0.30–0.64)
ST	0.50 (0.08)	(0.32–0.71)

**Figure 1:**
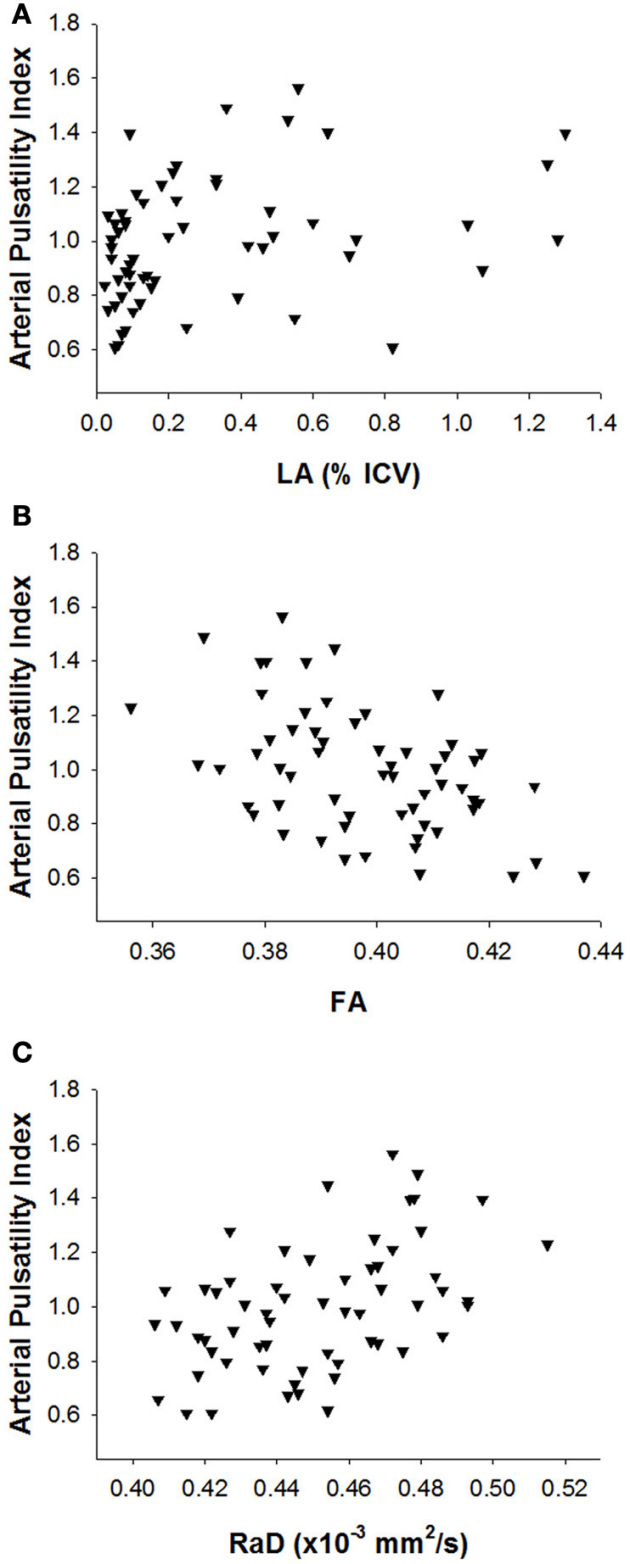
**Scatterplots of the relationship between arterial pulsatility index and (A) leukoaraiosis severity, (B) fractional anisotropy, and (C) radial diffusivity**.

Table 3 presents correlations between white matter measures and pulsatile haemodynamic flow measures. Arterial pulsatile measures showed a consistent pattern of association with measures of white matter disruption. LA severity showed a significant positive correlation with arterial pulse wave amplitude, but this correlation was not retained when controlling for age (Table 3B). Microstructural white matter measures showed stronger findings than LA severity. All arterial pulsatility measures were significantly correlated with both mean FA and RaD (Figure 1). Specifically, higher arterial pulsatility index (Table 3A), larger arterial pulse wave amplitude (Table 3B) and smaller arterial pulse width (Table 3C) were associated with lower FA and higher RaD scores and all correlations remained significant after controlling for age.

**Table 3:** **Partial correlations between measures of white matter disruption and measures of pulsatile haemodynamics, correlations in bold were significant after correcting for age**.

	**Arterial**	**SSS**	**ST**
**(A)PULSATILITY INDEX**			
LA	–	–	–
FA	−0.483^***^	–	−0.332^*^
RaD	0.467^**^	–	0.333^*^
**(B) PULSE WAVE AMPLITUDE**			
LA	0.337^*^	–	–
FA	−0.505^***^	–	–
RaD	0.510^***^	–	–
**(C) PULSE WIDTH**			
LA	–	–	–
FA	0.387^*^	–	–
RaD	−0.398^**^	–	–

Abbreviations: LA, leukoaraiosis severity; FA, fractional anisotropy; RaD, radial diffusivity; SSS, superior sagittal sinus; ST, straight sinus.

* *p < 0.05*,

** *p < 0.01*,

*** p < 0.001 (after Bonferroni correction).

None of the venous pulsatile measures for the SSS region was significantly correlated with measures of white matter disruption (Table 3). Increased pulsatility index for the deep ST venous region was significantly associated with reduced FA and increased RaD (Table 3A). However, the correlation between ST pulsatility and RaD was eliminated after controlling for age. Moreover, the correlation between ST pulsatility and FA was eliminated when controlling for both age and corresponding arterial pulsatility (*p* > 0.463).

As shown in Table 3, all three measures of arterial pulsatility (pulsatility index, pulse wave amplitude and pulse width) were associated with both FA and RaD, but FA was slightly more sensitive after adjusting for age. However, since age was correlated with LA severity (*r* = 0.409) and both age and LA severity were associated with FA (*r* = −0.387 and *r* = −0.405), we performed a multiple linear regression using age, LA severity and the arterial pulsatility index as explanatory variables with FA as the dependent variable. This analysis resulted in two models. The first model included only arterial pulsatility index as a predictor of FA (*R* = 0.483, adjusted *R*^2^ = 0.220), while the second included both arterial pulsatility index and LA severity as predictors of FA (*R* = 0.554, adjusted *R*^2^ = 0.283). Age was not a significant predictor of FA in either model. These findings suggest that arterial pulsatility is a stronger predictor of white matter FA than either age or LA severity. The results were replicated in separate analyses using arterial pulse wave amplitude and arterial pulse width instead of arterial pulsatility index as one of the predictors^1^.

**Figure 2:**
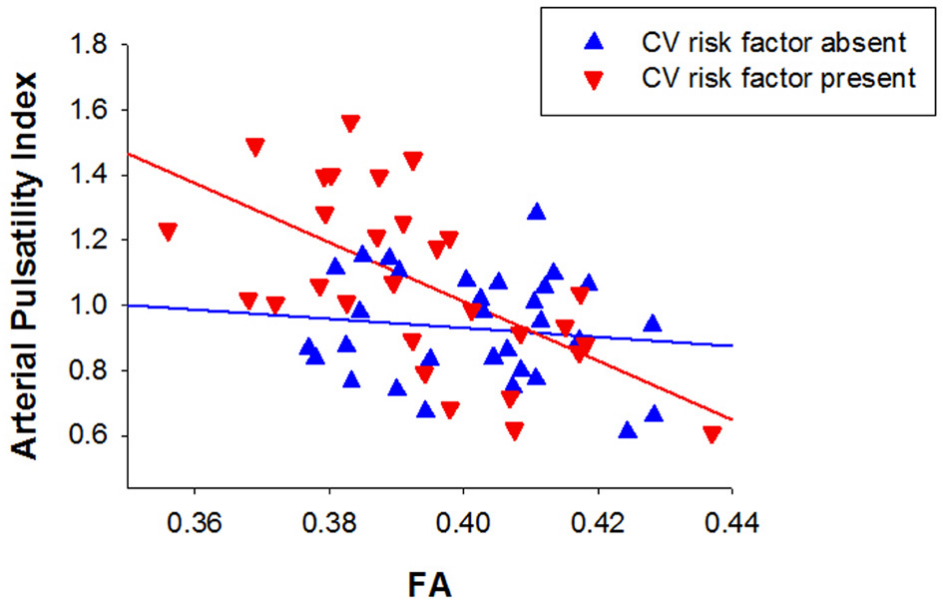
**Relationship between arterial pulsatility index and fractional anisotropy for participants with (triangles down) and without cardiovascular risk factors (triangles up)**.

The presence of cardiovascular risk factors (e.g., hypertension, hypercholesterolemia, atrial fibrillation, diabetes) is likely to influence both blood flow pulsatility and microstructural changes to the white matter and to be associated with greater risk for LA. Figure 2 depicts the data presented in Figure 1B coded on the basis of presence or absence of cardiovascular risk factors. Arterial pulsatility was strongly correlated with FA in participants with one or more cardiovascular risk factor (*r* = 0.614, *p* < 0.001), but not in participants with no cardiovascular risk factors (*r* = −0.127, *p* = 0.248). Thus, the association between white matter disruption and increased arterial pulsatility appears to be influenced by the presence of cardiovascular risk factors.

To summarize, measures of arterial and venous non-pulsatile haemodynamics were not significantly related with any measure of white matter disruption. Measures of white matter microstructure (e.g., FA and RaD) were weakly correlated with aqueduct pulse volume and with pulsatility in deep venous territories, but more strongly correlated with measures of arterial pulsatile haemodynamics. While macrostructural changes in white matter (i.e., LA severity) were also correlated with arterial pulsatility, the correlation was notably weaker than for white matter microstructure and failed to remain significant after controlling for age. Together these findings suggest that disruption to white matter at a microstructural level is associated with greater inflow pulsatility.

## Discussion

This is the first study to show that measures of pulsatile haemodynamics in older adults with negligible or mild LA are associated with microstructural changes in the white matter. Specifically, increased pulsatility index, larger pulse wave amplitude and narrower pulse width for arterial blood flow were significantly associated with lower FA and higher RaD, indicative of microstructural disruption of white matter organization. These findings indicate that the reduction in white matter FA often seen with increasing age is linked to the age-related increase in arterial pulsatility and highlight the potential role of arterial pulsatility in explaining age-related reduction in FA. Measures of pulsatile haemodynamics in deep venous territory (i.e., straight sinus) were also associated with lower FA and higher RaD. However, these relationships were weak and were not retained when controlling for the corresponding arterial measures.

Increased arterial pulsatility, as indicated by a higher pulsatility index, larger pulse wave amplitude and narrower pulse width, results in increased pulse energy. This increase in pulse energy may lead to a breakdown in Monroe-Kellie homeostasis and disrupt vascular and perivascular ultrastructure (Bateman, 2004). Increased arterial pulsatility is indicative of a decrease in arterial compliance and has been suggested to be due to cerebral arteriosclerosis (Henry-Feugeas, 2009). If this increased energy is not effectively dampened, it may over time filter into deep venous territories, resulting in remodeling of these venous structures (venous collagenosis) and reduced compliance in these vessels. This could result in obstruction of venous outflow channels and the reflection of pulse wave energy from the venous structures back toward the capillary bed. In turn, this may intensify capillary pulsation and perivascular sheer stresses, promoting further perivascular demyelination. This model fits well with the current findings that arterial pulsatility was more consistently related to extent of microstructural damage to the white matter whereas the venous findings were weaker and mediated by arterial changes. However, as these findings are based on correlational analyses, experimental studies are required to test this model.

In the present study, we showed that LA severity was weakly associated with arterial pulsatility, but this relationship was mediated by variability related to age. This finding is somewhat discrepant with the findings of earlier studies (Bateman, 2002; Doepp et al., 2006; Bateman et al., 2008; Mitchell et al., 2011; Webb et al., 2012) that tested participants with moderate to marked LA severity. As our participants had only negligible or mild LA, the weak correlation between vascular pulsatility and LA severity may be due to a restriction of variability in LA severity. Henry-Feugeas et al. (2009) also showed that increased arterial pulsatility is not a necessary condition for the onset of LA, suggesting that reduced intracranial compliance even under normal/low arterial pulsation conditions may be an alternative main determinant of LA severity. Together these findings suggest two possible mechanisms to LA: an “arteriosclerotic” process associated with severe arterial windkessel dysfunction, and a “resistive pulse wave encephalopathy” process associated with a decrease in craniospinal compliance.

Importantly, the current findings show that in people with mild LA, pulsatile components of blood flow are more closely related to white matter microstructure than macrostructure. Bateman et al. (2008), see also Henry-Feugeas and Koskas, 2012) suggested that increased vascular pulsatility may cause white matter damage by increasing perivascular shear stress and inducing perivascular atrophic demyelination. FA is posited as an indicator of microstructural integrity (Basser and Jones, 2002), whereas RaD is affected by variability in myelination (Song et al., 2005). These measures are therefore likely to be sensitive to early changes in the structural organization of white matter that are likely to precede the emergence of LA.

It is important to note that we used measures of mean FA and mean RaD across the whole brain as estimates of diffuse microstructural properties of white matter. Our reasoning was that since arterial pulsatility is calculated at the level of arterial flow into the cranial cavity, it would be useful to examine whether this is associated with changes in whole brain white matter. However, measures of FA and RaD are influenced by microstructural tissue properties, such as myelin density and membrane integrity, which vary throughout the brain. Thus, some white matter regions may be more susceptible to changes associated with increased arterial pulsatility than others. We examined this by tested the relationship between arterial pulsatility and FA / RaD in 18 prominent white matter tracts derived using the John Hopkins University (JHU) white matter tract atlas. While arterial pulsatility was significantly associated with both FA and RaD in 17 of the 18 white matter tracts (*p* < 0.041, with 14 of these having *p* < 0.003), it was most strongly correlated with whole brain FA and RaD than for any specific white matter tract. It is likely that microstructural white matter measures in specific tracts will be more strongly associated with measures of arterial pulsatility in smaller arteries that supply these relevant brain regions. However, importantly, the present findings suggest that whole brain FA and RaD may be sensitive measures of early changes in white matter microstructure as a result from changes in pulse-wave dynamics that emerge prior to the manifestation of gross macrostructural changes on T2-weighted MR images.

### Cardiovascular risk factors

The association between arterial pulsatility and white matter microstructure was only present for participants who presented with one or more cardiovascular risk factor. While the analysis was *post-hoc*, the results support the contention that white matter disruption associated with increased arterial pulsatility in participants with negligible or mild LA is influenced by the presence of cardiovascular risk factors. Although this finding is intriguing, it is possible that it is partly due to greater variation in both arterial pulsatility and FA in the presence of cardiovascular risk factors. Further work is needed to confirm this relationship and investigate the effect of specific risk factors.

### Non-pulsatile haemodynamic findings

As predicted, we found no relationship between LA severity and measures of arterial inflow in older adults with mild LA and no evidence of dementia. This is consistent with previous findings (Bateman, 2002; Henry-Feugeas et al., 2009) and provides further evidence that LA may occur independently of ischaemia or reduced arterial flow. Bateman et al. (2008) showed that SSS outflow as a percentage of inflow was reduced in patients with vascular dementia and LA, when compared to a healthy ageing group. Reduction in SSS outflow has been shown to be related to increased SSS pressure, resulting in a pressure differential between superficial and deep venous territories (Bateman, 2007). This is thought to promote pulsation redirection to the deep system as seen in vascular dementia patients. In our healthy older adults with only mild LA, we found no relationship between LA severity and SSS venous return. These findings suggest that elevated SSS venous pressure and reduced SSS flow is evident in severe but not mild LA.

### Cerebral hydrodynamic findings

It is interesting that neither CSF pulse volume nor the index of intracranial compliance showed an association with LA severity despite both being associated with FA and RaD. This detectable change in CSF flow in the aqueduct is thought to result from increased passage of the systolic pulse wave into the capillary bed, thereby increasing capillary pulsation (Stivaros and Jackson, 2007). During capillary pulsation, brain expansion is directed toward the ventricles, which results in an increase in intraventricular pulse pressure and hyperdynamic flow of CSF through the cerebral aqueduct, as described in hydrocephalic patients (Henry-Feugeas et al., 2005). In addition to increased CSF flow through the cerebral aqueduct, increased pulsations within the ST was also associated with FA and RaD. Thus, as seen previously in patients with vascular dementia (Bateman et al., 2008), increased pulsations are preferentially distributed to the ST territory and CSF outflow. This provides further evidence that larger arterial pulsations in the deep parenchyma of the brain are associated with demyelination and perivascular rarefaction.

## Conclusion

The present study showed that, in older adults with negligible or mild LA and no evidence of dementia, increased arterial pulsatility is related to changes in white matter microstructural organization in the absence of an association with LA severity. These findings suggest that pulse wave injury is associated with early disruption to the structural properties of white matter. We argue that changes in white matter microstructure measured through DTI may provide an early marker of damage to the perivascular white matter arising as a result of pulse wave injury well before this injury results in LA. While intriguing, these conclusions are based on correlational analyses and hence can only speculate regarding causation. Prospective longitudinal designs are needed to examine whether arterial pulsatility and microstructural white matter measures can predict future severity of LA.

### Conflict of interest statement

The authors declare that the research was conducted in the absence of any commercial or financial relationships that could be construed as a potential conflict of interest.

## Footnotes

^1^Arterial pulse wave amplitude was the strongest predictor of mean FA (*R* = 505, adjusted *R*^2^ = 0.242) and model was significantly improved when LA severity was also included (*R* = 0.563, adjusted *R*^2^ = 0.293). When using arterial pulse width, LA severity was the stronger predictor of FA (*R* = 405, adjusted *R*^2^ = 0.150), and the model was significantly improved when adding arterial pulse width (*R* = 515, adjusted *R*^2^ = 0.239).
